# Advances in Research on Nutrition and Childhood Obesity

**DOI:** 10.3390/children10010022

**Published:** 2022-12-23

**Authors:** George Antonogeorgos

**Affiliations:** 1 Department of Nutrition and Dietetics, School of Health Sciences and Education, Harokopio University, 70 Eleftheriou Venizelou (Thiseos) Ave. Kallithea, 17671 Athens, Greece; gantonogeorgos@gmail.com; 2 Senior Research Associate in Pediatric Pulmonology 3rd Department of Paediatrics, National and Ka-Podistian Athens University, Univercity General Hospital “ATTIKON”, Rimini 1, Chaidari, 12462 Athens, Greece

Obesity is a complex, multifactorial problem affecting children and adolescents around the world. It has been implicated as a critical factor in the development of many childhood diseases, such as Type 2 Diabetes Mellitus (DM), hypertension and Non-Alcoholic Fatty Liver Disease (NAFLD), or as a co-morbidity in others, such as hypothyroidism and Prader-Willi syndrome. The study of the role of lifestyle factors has gained the attention of researchers in recent years, since these are modifiable and could be easily involved in preventive interventions. Among them, nutrition is one of the most crucial. Whether as micro or macronutrients intake or as dietary patterns, in recent decades, diet has emerged as a key factor in the well-being of children.

In the Special Issue (SI) “Advances in Research on Nutrition and Childhood Obesity”, researchers from around the world were invited to contribute novel evidence concerning the role of nutrition in childhood obesity. As a result, I am pleased to present a plethora of original research on different topics and pediatric populations. In this Special Issue, novel knowledge about the feeding of preterm neonates was revealed by Sokou et al., who evidenced that continuously feeding them with milk had no gastrointestinal complications or adverse effects compared with the current clinical practice of intermittent feeding [1]. Nutrition and obesity in the context of chronic childhood diseases were also present in the topics of the SI. Nsamba et al., who reported higher fat accumulation in Type 1 DM children than in their non-DM counterparts, while abdominal fat accumulation was associated with poorer glycemic control in these patients [2]. Moreover, Ahn et al. proposed that the use of Fetuin A-to-Adiponectin ratio could be useful in identifying diabetic obese children and adolescents with a higher probability of developing metabolic complications [3] Two articles from the Greek division of the Global Asthma Network presented important evidence about the protective association between a high consumption of fruits and vegetables with atopic diseases and the important role of socioeconomical status in this relationship [4,5]. Obesity also enhances inflammation, and the manuscript by Martin-Hadmas et al. reported an important positive correlation between weight status and interleukins 6 and 8 in healthy children aged 6 to 12 years [6]. Micronutrient and vitamin intake plays an important role in childhood growth and development, and two of the most important ones were investigated in two special populations: vitamin D and iron. The majority of Swiss obese children attending a reference center for the treatment of obesity were lacking vitamin D, while iron deficit intake was reported for Moroccan children living in the mountainous aeras of their country [7,8]. Most interesting, milk consumption, a food rich in vitamin D, was reported to be inversely related to obesity in preadolescents, enriching the current literature and highlighting the significant contribution of vitamin D in weight regulation [9]. On the other hand, excess body weight was found to be positively associated with cardiometabolic risk factors, especially triglycerides in school-aged children living in an urban environment [10]. Factors such as mothers with depressive symptomatology were also examined and found to have no association with the Body Mass Index (BMI) of their offspring, regardless of the duration of breastfeeding [11]. A further two manuscripts examined the association of weight status with two clinical outcomes: idiopathic scoliosis and antihistamine intake. A positive association between antihistamines—a commonly used medicine in pediatric clinical practice and BMI—was reported, while low BMI was related with the higher presence of spinal deformity and idiopathic scoliosis in adolescents [12,13]. Finally, a very important inverse association was reported between breakfast intake and overweight/obese adolescents in European adolescents aged 12.5 to 17.5 years old [14]. Thus, every clinical or public health worker implicated in the care of children should keep in mind these important results.

Research on micronutrient and macronutrient dietary intake, as well as food patterns and their relation to various aspects of children’s lives is also present in this SI. The changes in parental lifestyle habits and their correlations with the changes in their children’s diet during the first COVID-19 Lockdown in Greece were presented in the paper by Saltaouras et al [15] Evidence was also reported by Almazan et al. [16] regarding the highest risk for Candida spp colonization in preschool Mexican aged children who adhered to a diet high in simple carbohydrates. Moreover, the manuscript by Sari et al. offer important input about the association of nutrient intake and quality of life in girls living in a rural area in Indonesia, a population which is prone to malnutrition and poverty [17]. Finally, one systematic review summarized evidence about the role of family in the association between obesity and depression in children, reporting the lack of studies in this area, while one meta-analysis studied the effect of air pollution on childhood obesity and found strong evidence about its harmful effect on the risk of developing overweight or obesity in children [18,19]. Finally, the role of nutrition in the prevention of diet-related diseases was explored in children and young adults with Down Syndrome [20].

This SI of the journal Children aimed to provide novel enlightenment on several topics in the fields of nutrition and obesity in childhood, as well as updated evidence about their interrelation and their relations to several children’s health and wellbeing factors. The high-quality published manuscripts reassure that scholars involved in pediatric research can find fruitful and useful knowledge to assist them in their continuing effort to understand the association between nutrition and obesity in childhood.

## References

[1] Sokou R., Grivea I.N., Gounari E., Panagiotounakou P., Baltogianni M., Antonogeorgos G., Kokori F., Konstantinidi A., Gounaris A.K. (2021). Gastric Volume Changes in Preterm Neonates during Intermittent and Continuous Feeding-GRV and Feeding Mode in Preterm Neonates. Children.

[2] Nsamba J., Eroju P., Drenos F., Mathews E. (2022). Body Composition Characteristics of Type 1 Diabetes Children and Adolescents: A Hospital-Based Case-Control Study in Uganda. Children.

[3] Ahn M.B., Kim S.K., Kim S.H., Cho W.K., Suh J.S., Cho K.S., Suh B.K., Jung M.H. (2021). Clinical Significance of the Fetuin-A-to-Adiponectin Ratio in Obese Children and Adolescents with Diabetes Mellitus. Children.

[4] Antonogeorgos G., Priftis K.N., Panagiotakos D.B., Ellwood P., García-Marcos L., Liakou E., Koutsokera A., Drakontaeidis P., Moriki D., Thanasia M. (2021). Exploring the Relation between Atopic Diseases and Lifestyle Patterns among Ado-lescents Living in Greece: Evidence from the Greek Global Asthma Network (GAN) Cross-Sectional Study. Children.

[5] Antonogeorgos G., Priftis K., Panagiotakos D., Ellwood P., García-Marcos L., Liakou E., Koutsokera A., Drakontaeidis P., Thanasia M., Mandrapylia M. (2021). Parental Education and the Association between Fruit and Vegetable Consumption and Asthma in Adolescents: The Greek Global Asthma Network (GAN) Study. Children.

[6] Martin-Hadmas R.M., Martin S.A., Romonti A., Marginean C.O. (2021). Anthropometric Development in Children: Possible Changes in Body Mass, Basal Metabolic Rate and Inflammatory Status. Children.

[7] Patriota P., Borloz S., Ruiz I., Bouthors T., Rezzi S., Marques-Vidal P., Hauschild M. (2022). High Prevalence of Hypovitaminosis D in Adolescents Attending a Reference Centre for the Treatment of Obesity in Switzerland. Children.

[8] El Ammari L., Saeid N., Talouizte A., Gamih H., Labzizi S., Mendili J.E., Rami A., Idrissi M., Benjeddou K., Zahrou F.E. (2021). A Household-Based Survey of Iodine Nutrition in Moroccan Children Shows Iodine Sufficiency at the National Level But Risk of Deficient Intakes in Mountainous Areas. Children.

[9] Kanellopoulou A., Kosti R.I., Notara V., Antonogeorgos G., Rojas-Gil A.P., Kornilaki E.N., Lagiou A., Yannakoulia M., Panagiotakos D.B. (2022). The Role of Milk on Children’s Weight Status: An Epidemiological Study among Preadolescents in Greece. Children.

[10] Bhagavathula A.S., Al-Hamad S., Yasin J., Aburawi E.H. (2021). Distribution of Cardiometabolic Risk Factors in School-Aged Children with Excess Body Weight in the Al Ain City, United Arab Emirates: A Cross-Sectional Study. Children.

[11] Pineros-Leano M., Saltzman J.A., Liechty J.M., Musaad S., Aguayo L. (2021). Maternal Depressive Symptoms and Their Association with Breastfeeding and Child Weight Outcomes. Children.

[12] Jeon K.K., Kim D.I. (2021). Low Body Mass Index Levels and Idiopathic Scoliosis in Korean Children: A Cross-Sectional Study. Children.

[13] Saad M., Syed S., Ilyas M., Gashev A.A. (2020). Antihistamines Increase Body Mass Index Percentiles and Z-Scores in Hispanic Children. Children.

[14] Cacau L.T., De Miguel-Etayo P., Santaliestra-Pasias A.M., Gimenez-Legarre N., Marchioni D.M., Molina-Hidalgo C., Censi L., Gonzalez-Gross M., Grammatikaki E., Breidenassel C. (2021). Breakfast Dietary Pattern Is Inversely Associated with Overweight/Obesity in European Adolescents: The HELENA Study. Children.

[15] Androutsos O., Perperidi M., Georgiou C., Chouliaras G. (2021). Lifestyle Changes and Determinants of Children’s and Adolescents’ Body Weight Increase during the First COVID-19 Lockdown in Greece: The COV-EAT Study. Nutrients.

[16] Pinto-Almazan R., Frias-De-Leon M.G., Fuentes-Venado C.E., Arenas R., Gonzalez-Gutierrez L., Chavez-Gutierrez E., Torres-Paez O.U., Martinez-Herrera E. (2022). Frequency of Candida spp. in the Oral Cavity of Asymptomatic Preschool Mexican Children and Its Association with Nutritional Status. Children.

[17] Sari P., Herawati D.M.D., Dhamayanti M., Hilmanto D. (2022). The Study of Nutrient Intake and Adolescent Girls’ Quality of Life in a Rural Area of Indonesia. Children.

[18] Kanellopoulou A., Antonogeorgos G., Douros K., Panagiotakos D.B. (2022). The Association between Obesity and Depression among Children and the Role of Family: A Systematic Review. Children.

[19] Parasin N., Amnuaylojaroen T., Saokaew S. (2021). Effect of Air Pollution on Obesity in Children: A Systematic Review and Meta-Analysis. Children.

[20] Białek-Dratwa A., Żur S., Wilemska-Kucharzewska K., Szczepańska E., Kowalski O. (2023). Nutrition as Prevention of Diet-Related Diseases—A Cross-Sectional Study among Children and Young Adults with Down Syndrome. Children.

